# Supplementary material for: WordCommentsAnalyzer: A windows software tool for qualitative research
Source: F1000Res. 2018 Sep 4;7:536. Originally published 2018 May 3. [Version 2] doi: 10.12688/f1000research.14819.2 (PMC6137411; doi:10.12688/f1000research.14819.2)
Supplement: Supplementary file 3 [file f1000research-7-17614-s0002.tgz › b2a79496-4ada-47c4-9682-a99ce867836d.zip!/Eight Iranian Health Journals/The Journal of Toloo-e-Behdasht_35.docx]

© The Journal of Toloo-e-Behdasht. This work is licensed under CC BY 4.0 (https://creativecommons.org/licenses/by/4.0/).

Volume & Issue: دوره 11 - شماره 1

Title: حذف نیکل از آب خنک‌کننده مصنوعی نیروگاه اتمی با استفاده از نانوذرات آهن اکسید

http://tbj.ssu.ac.ir/article-1-84-fa.html

سابقه و اهداف: خنک کننده اولیه یک بستر خنک کنندگی ضروری برای کنترل حرارت در نیروگاه‫های هسته‫ای است. در طی فرآیند اکسیداسیون، نیکل می­تواند در خنک­کننده اولیه آزاد شود که آلودگی آنرا افزایش می­دهد. مشکلات بهره­برداری و هزینه بالای تصفیه آب خنک­کننده در روش­های موجود، تحقیقات برای روش­های جدیدتر را ضروری می­سازد. هدف از انجام این تحقیق بررسی حذف Ni(II) از محیط حقیقی آب خنک­کننده مصنوعی نیروگاه اتمی با استفاده از نانوذرات آهن اکسید و پارامترهای موثر بر آن می­باشد. روش بررسی: در این پژوهش نانوذرات آهن صفر ظرفیتی به نانوذرات آهن اکسید تبدیل شد و کارایی حذف آن برای یون Ni(II) در آب خنک­کننده مصنوعی ناپیوسته مورد مطالعه قرار گرفت. هم­چنین تاثیر عوامل موثر در فرآیند حذف شامل غلظت نانوذرات آهن اکسید، pH محلول و زمان تماس بررسی و ایزوترم جذب و مدل سینتیکی واکنش معین گردید. یافته­ها: تصاویر STM نشان داد که اندازه ذرات نانو ذرات اکسید آهن کمتر از nm 40 می­باشد. در pH اولیه محلول و h 4 = t حذف تقریبا کامل یون نیکل در غلظت g/L 40 جاذب بدست آمد. در g/L 1 = C جاذب و h 4 = t، بیشترین راندمان حذف در 10 = pH بود. جذب نیکل بر نانوذرات آهن اکسید در            g/L 35 = C جاذب و 71/2 = pH از ایزوترم لانگمیر تبعیت می­کرد و سینتیک واکنش شبه درجه دوم بود.  نتیجه­گیری: نتایج این مطالعه نشان می­دهد که با افزایش pH در محیط قلیایی راندمان حذف افزایش می­یابد. زیرا وقتی که pH محلول بالای نقطه ایزوالکتریک باشد، سطح اکسید به طور منفی شارژ می­شود و می­تواند با کاتیون­ها (مثلا یون­های فلزی) تشکیل کمپلکس­های سطحی دهد. با اعمال شرایط بهینه نانو ذرات آهن اکسید توانایی خالص سازی آب خنک­کننده را دارد.

Title: بررسی وضعیت ویتامین D در بیماران مبتلا به افسردگی مراجعه کننده به کلینیک های تخصصی وابسته به دانشگاه علوم پزشکی یزد 1390

http://tbj.ssu.ac.ir/article-1-85-fa.html

سابقه و اهداف: نقش ویتامین D در ارتباط با بیماری هایی مثل استئوپروز، سرطان، بیماریهای قلبی عروقی و دیابت درمطالعات مختلف شناخته شده و اخیراً نقش این ویتامین درعملکرد مغز، مثل عملکردهای شناختی و سلامت روانی مطرح گشته است. مطالعات نشان داده اند غلظت ویتامین D در بیماران افسرده و مبتلا به اختلالات روانی پایین است و ممکن است بین اختلالات روانی و کمبود ویتامین D رابطه ای وجود داشته باشد. این مطالعه به منظور بررسی وضعیت ویتامین D در بیماران مبتلا به افسردگی مراجعه کننده به پلی کلینیک های وابسته به دانشگاه علوم پزشکی یزد انجام شده است.   روش بررسی: در این مطالعه توصیفی- مقطعی 211 نفر بیمار افسرده که نمره تست بک بالای 17 داشتند شرکت کردند. بعد از تکمیل پرسشنامه افسردگی بک سایر داده های دموگرافیک بیماران ثبت و 5 سی سی نمونه خون ناشتا برای تعیین غلظت سرمی 25-هیدروکسی ویتامین D گرفته شد. غلظت زیر 35 نانو مول بر لیتر به عنوان کمبود ویتامین D در نظر گرفته شد.   یافته ها: از بین 211 فرد شرکت کننده،70 نفر(2/33 درصد) مرد و141 نفر(8/66 درصد) زن بودند. میانگین سنی افراد 4/33 سال بدست آمد. میانه 25-هیدرکسی ویتامین D در کل، مردان و زنان به ترتیب 4/26، 36 و 6/22 نانو مول در لیتر بود. 3/34 درصد از افراد وضعیت ویتامین D طبیعی داشتند و 7/65 درصد افراد به درجاتی از کمبود ویتامین D مبتلا بودند. 6/7 درصد از افراد افسرده از کمبود شدید، 5/39 درصد از کمبود متوسط و 6/18 درصد از کمبود خفیف ویتامین D رنج می بردند. بین وضعیت ویتامین D و شدت افسردگی ارتباط معنی داری بدست نیامد.   نتیجه گیری: با توجه به نتایج این مطالعه شیوع کمبود ویتامین D در میان افراد افسرده مثل سایر طبقات جمعیتی در منطقه مورد مطالعه، بالاست و نیازمند مطالعه بیشتر در خصوص شناسایی علت آن و نیز بررسی اثر رفع کمبود آن بر وضعیت افسردگی می باشد.

Title: بررسی اپیدمیولوژی حیوان گزیدگی و علل تاخیر در درمان پیشگیرنده در شهرستان طبس بین سال‌های 1389-1385

http://tbj.ssu.ac.ir/article-1-86-fa.html

سابقه و اهداف: هاری از جمله بیماری‌های کشنده در انسان و دیگر پسنانداران است. با توجه به عدم کنترل کامل بیماری در حیوانات اهلی و وحشی در کشور ما هنوز هم احتمال بروز بیماری وجود دارد. هدف این مطالعه بررسی همه‌گیر شناسی هاری و حیوان گزیدگی و علل تاخیر در گرفتن درمان پیشگرنده در شهرستان طبس طی سال‌های 1389-1385 می‌باشد.   روش بررسی: پژوهش حاضر از نوع توصیفی مقطعی بوده که در آن از اطلاعات موجود 480 مورد فرد حیوان‌گزیده مراجعه کننده به واحدهای هاری شهرستان طبس در طی سال­های 1389-1385 استفاده گردید. فاکتورهای مربوط به بیمار، فاکتورهای مربوط به مکان زندگی، فاکتورهای مربوط به زمان گزیده شدن، وضعیت جراحت، محل گزش مورد بررسی قرار گرفتند. جهت تحلیل داده­ها، از آمار توصیفی شامل شاخص­های توصیفی، و از تحلیل رگرسیون لوجستیک جهت سنجش اثر متغیرهای مورد نظر بر تاخیر در دریافت درمان پیشگیرنده از هاری استفاده گردید. تحلیل به کمک نرم افزار SPSS نسخه 18 انجام گرفت.   یافته‌ها: از مجموع 480 مورد بررسی شده، 413 نفر (86%) مرد و 67 نفر (14%) زن بودند. 6/54% (262 نفر) حادثه حیوان گزیدگی را در شهر و 4/45% (218 نفر) آن را در روستا تجربه کرده بودند. بیشترین موارد گزش به ترتیب مربوط به سگ (1/63%) و گربه (3/27%) بوده است . بیشترین گزش در مشاغل، محصل 107 نفر (3/22%) و آزاد 98 نفر (4/20%) بود. میانگین و انحراف معیار زمان تاخیر در دریافت واکسن ضد هاری 8/1 ± 5/6 ساعت بوده است. مهمترین عوامل تاثیر‌گذار در داشتن یا نداشتن این تاخیر عبارت بودند از سن (98/0 OR= ، 99/0-74/0 CI: )، ساعت حادثه (06/1 OR= ، 12/1-01/1 CI: )، گزش اندام تحتانی (31/5 OR= ، 33/6-8/3 CI: )، سابقه واکسیناسیون (39/0 OR= ، 68/0-21/0 CI: )، داشتن مراقبت اولیه توصیه شده برای زخم (75/6 OR= ، 8/8-4/4 CI: ) و نوع حیوان (17/4 OR= ، 5/6-34/3 CI: ) .   نتیجه‌گیری: با توجه به میزان بروز نسبتا بالای حیوان‌گزیدگی در این شهرستان لزوم کنترل حیواناتی مانند سگ و گربه از طریق آموزش به قشر آسیب پذیر مانند نوجوانان و صاحبان آنان و هماهنگی بین بخشی برای از بین بردن حیوانات ولگرد بیش از پیش باید مورد توجه قرار گیرد. با توجه به هزینه تحمیل شده ناشی از هر مورد حیوان‌گزیدگی به سیستم مراقبت بهداشتی لازم است اقدامات پیشگیری کننده جهت جلوگیری از گزش در اولویت قرار گیرند.

Title: مقایسه تاثیر آموزش مهارت های ارتباطی به کارکنان بهداشتی به دو شیوه سخنرانی و موادچاپی بر رضایتمندی مراجعین آنان در مراکز بهداشتی درمانی شهرستان شیراز

http://tbj.ssu.ac.ir/article-1-87-fa.html

سابقه واهداف: یکی از مهمترین عوامل موثر بر افزایش رضایت مندی مراجعان ،نحوه ارتباط ارائه دهندگان خدمات بهداشتی و توانایی در برقراری ارتباط مناسب با مراجعین می باشد. با در نظر گرفتن این امر، مطالعه نیمه تجربی حاضر با هدف بررسی و مقایسه میزان تاثیر روشهای آموزش سخنرانی و نوشتاری به کاردانهای بهداشت خانواده بر رضایت مندی مراجعین آنها در مراکز بهداشتی درمانی به انجام رسید.   روش بررسی: تعداد 25نفر از کارکنان بهداشت خانواده و حدود 1000 مراجعه کننده در 6 مرکز در دو مرحله در این مطالعه شرکت داشتند.برای جمع آوری اطلاعات از پرسشنامه رضایت مندی مراجعان از نحوه ارتباط کارکنان بهداشت خانواده استفاده شد .جمع آوری اطلاعات در دو مرحله قبل و بعد از مداخله آموزش مهارت ارتباطی صورت گرفت . بعد از جمع آوری اطلاعات مرحله اول، آموزش به کارکنان در دو گروه 1- شیوه سخنرانی در دوره آموزشی یک روزه 2- شیوه نوشتاری بصورت ارائه جزوات آموزشی در محل کار ارائه شد . درمرحله دوم ، 6 هفته بعد از آموزش با استفاده از همان ابزار، اطلاعات جمع آوری شد وبا استفاده نرم افزار SPSS و تستهای آماری کای دو و رگرسیون مورد تجزیه و تحلیل قرار گرفت.   یافته ها: یافته های مطالعه نشان داد تغییر میانگین نمره رضایت مندی در گروه آموزش به شیوه سخنرانی از 47/37 در مرحله قبل از مداخله به 86/44 بعد ازمداخله آموزشی رسید که از نظر آماری معنادار بود (0001/0 p< ).تغییر میانگین نمره رضایت مندی در گروه آموزش به شیوه نوشتاری از 39 در مرحله قبل از مداخله به 5/40در مرحله بعد ازمداخله آموزشی رسید که از لحاظ آماری معنادار بود(016/0 p= ). دربررسی وضعیت رضایت مراجعین کارکنان آموزش دیده با شیوه سخنرانی ،میزان رضایت کامل از 8/29 درصد قبل از مداخله به 3/81 درصد بعداز مداخله رسیده است که این اختلاف آماری با (0001/0 p< ) معنی داربود.   نتیجه گیری : نتا یج حاصل بیانگر آن است که آموزش مهارتهای ارتباطی به کارکنان بهداشت خانواده بر رضایت مندی مراجعین تاثیر مثبت داشته است و آموزش سخنرانی تاثیر بسیار بیشتری داشته است .بنابراین پیشنهاد می شود برای افزایش رضایت مندی مراجعین و در نتیجه ارتقای نتایج مفید خدمات بهداشتی، دوره های آموزشی مهارتهای ارتباطی جهت کارکنان برگزار گردد.

Title: بررسی تأثیر فناوری اطلاعات و ارتباطات بر فرایند تعلیم و تربیت از دیدگاه دانشجویان دانشگاه یزد

http://tbj.ssu.ac.ir/article-1-88-fa.html

سابقه واهداف: نظام آموزش عالی به عنوان متولی اصلی دربخشهای آموزشی و پژوهشی و در راستای تعلیم و تربیت نیروهای متخصص و کارآمد هر کشور نقش داشته که با تاثیر پذیری از فناوریهای اطلاعات و ارتباطات نوین موجب ارتقاء سطح دانش عمومی و مهارت متخصصان در همه سطوح می گردد.ودر نهایت تأثیرات عمیقی بر زندگی انسانها داشته است که مهمترین و عمیق ترین این تأثیرات در زمینه های آموزش و یادگیری و به عبارتی تعلیم و تربیت می باشد. روش بررسی : پژوهش حاضر از نوع توصیفی- تحلیلی مقطعی می باشد و بر روی 406 نفر از دانشجویان دانشگاه یزد که به روش تصادفی طبقه ای انتخاب شده اند انجام گردید. ابزار جمع آوری داده ها پرسسشنامه محقق ساخته بودکه روایی پرسشنامه توسط متخصصین مربوطه تائید و پایایی آن با استفاده از ضریب آلفای کرونباخ 827. محاسبه گردید. پس از گردآوری داده ها با استفاده از نرم افزارspss. به منظور توصیف داده هااز شاخص های فراوانی ، درصد ، میانگین و انحراف معیارو برای آزمون فرضیه ها از آزمون های تی تست ، Anova یک طرفه ،کای اسکوار و آزمون همبستگی پیرسون استفاده گردید. یافته ها: پس از تجزیه وتحلیل داده ها مشخص گردید که 2/39 درصد دانشجویان کمتر از یک ساعت و 8/60 درصد بیشتراز یک ساعت درروز از تلویزیون استفاده می کنند و 7/58 درصد کمتر از یک ساعت و 3/41 درصد بیشتراز یک ساعت در روز از اینترنت استفاده نمودند. از دیدگاه آنان اعتیاد آورترین وسایل ارتباط جمعی به ترتیب اینترنت، تلفن همراه،ماهواره، تلویزیون، کتاب و رادیوبیان گردید. همچنین بین استفاده از فناوری اطلاعات و ارتباطات با رشد تعلیم و تربیت در دانشجویان رابطه معناداری وجود دارد (001/0p ).وبین هیچکدام از متغیرهای دموگرافیک (سن ،جنس،وضعیت تاهل ،شغل پدر ،شغل مادر،محل سکونت وگروه تحصیلی )با استفاده ازفناوری اطلاعات و ارتباطات رابطه معناداری وجود ندارد. نتیجه گیری: در این مطالعه فناوری اطلاعات و ارتباطات و وسائل ارتباط جمعی بر تعلیم و تربیت تاثیر گذاری بسیار بالایی داشته که نهایتا در روابط اجتماعی آحاد جامعه تأثیر گذاشته و امکان یادگیری را به صورت مستقل افزایش می دهد.لذا با بکارگیری فناوری اطلاعات و ارتباطات از ابزاری که قبلا برای جمع آوری و ذخیره دانش استفاده می شد به وسیله ای برای تولید دانش تغییر کرده و موجب پیدایش محیط دانش گرا می گردد.که خود فرایند تدریس و یادگیری را تسهیل و درنتیجه موجب رشد تعلیم و تربیت افراد می شود. واژه های کلیدی: فناوری اطلاعات و ارتباطات، تعلیم و تربیت ، دانشجویان

Title: مقایسه اثر ضدعفونی کنندگی اشعه ماوراءبنفش و ماده شیمیایی کورنکس بر سطوح اتاقهای عمل بیمارستان سوختگی شهید صدوقی یزد

http://tbj.ssu.ac.ir/article-1-89-fa.html

سابقه و اهداف : برای جلوگیری از پخش عوامل بیماری زا در محیط بیمارستان روشهای بهداشتی مناسبی برای ضدعفونی سطوح آلوده لازم است. یکی از روشها استفاده از مواد شیمیایی است که وقت گیر و زحمت دار بوده و برای کارکنان و محیط هم مضر است. تحقیقات در مورد روشهای مختلف ضدعفونی سالهای زیادی است که انجام می شود. نور UVC (100 تا 280 نانومتر) برای ضد عفونی آب، روی سطوح و هوا استفاده می شود ولی دوز کشنده لازم برای میکروبهای مختلف متفاوت است. در این تحقیق دوز حاصل از لامپهای UVC موجود در اتاقهای عمل بیمارستان سوختگی شهید صدوقی یزد (با طول موج nm 254) اندازه گیری و میزان ضد عفونی کنندگی آن با ماده شیمیایی کورنکس مقایسه گردید.   روش بررسی: این مطالعه به روش توصیفی مقطعی انجام گرفت. میزان تابش رسیده به قسمتهای مختلف اتاق با تقسیم بندی کف اتاقها بفواصل Cm 50 در ارتفاع 80 سانتیمتر بعد از 14 ساعت روشن بودن لامپها اندازه گیری شد. همچنین میزان تابش UVC در محل های خاصی که از پیش تعیین شده بود(15 محل) مانند روی میز، روی تخت، داخل قفسه و ... نیز اندازه گیری شد. نمونه های میکروبی از سطوح اتاقهای عمل قبل و بعد از تمیز کردن استاندارد بیمارستان، تابش UVC و ضدعفونی کننده کورنکس برداشته شد. نمونه ها با استفاده از پلیت های تماسی استاندارد جمع آوری شده و با یکدیگر مقایسه گردیدند.   یافته ها: نتایج نشان داد که دوز رسیده به قسمتهای مختلف کف اتاقهای عمل بین صفر تا 37951 ژول بر متر مربع بوده است. آزمایشات نشان داد که UVC تعداد کلونی ها را روی سطوح اتاق های عمل ، از متوسط cfu/sample 80/64 به cfu/sample 67/41 تقلیل میدهد و بعد از ضدعفونی اضافی شیمیایی با کورنکس به cfu/sample 53/16 کاهش پیدا میکند. بنابراین مشخص گردید که استفاده از UVC و ضدعفونی کننده کورنکس به تنها یی و یا در ترکیبهای مختلف تعداد کولونی های سطحی را بطور معنی داری کاهش می دهند( برای هر دو 001/0 P< ).   نتیجه گیری: با توجه به نتایج بدست آمده مشخص شد که در حداقل نیمی از اتاق میزان دوز تقریبا صفر بوده ولی در نزدیکی لامپها بعلت طولانی بودن زمان استفاده ، میزان دوز حتی از میزان دوز لازم برای غیرفعال سازی مقاومترین ویروسها بیشتر است. به این ترتیب نتیجه گیری شد که بایستی در تعداد و محل نصب و زمان روشن بودن لامپهای ضدعفونی کننده UVC تجدید نظر بعمل آید و متناسب با دوز لازم استاندارد برای تمام نقاط اتاق تنظیم گردد. از طرف دیگر UVC برای ضدعفونی سطوح کاملا در سایه مانند زیر تخت موثر نیست. بنابراین نور UVC بایستی بهمراه ضدعفونی کننده های شیمیایی مورد استفاده قرار گیرد.

Title: مقایسه تاثیر آموزش تغذیه توسط همتایان و پرسنل بهداشتی بر آگاهی، نگرش و شاخصهای تغذیه ای زنان 35-18 ساله تحت پوشش مراکز بهداشتی درمانی شهرستان ارومیه

http://tbj.ssu.ac.ir/article-1-90-fa.html

سابقه و اهداف: عدم آگاهی تغذیه ای و در نتیجه عملکرد نامناسب باعث مشکلاتی از قبیل سوء تغذیه و ابتلا به بیماری ها می شود . مطالعات اندکی در زمینه تأثیر روشهای مختلف آموزش تغذیه بر آگاهی، نگرش و شاخص های تغذیه ای زنان انجام گرفته است. لذا این پژوهش با هدف مقایسه تاثیر آموزش توسط همتایان و پرسنل بهداشتی بر آگاهی، نگرش و شاخص­­­­­­­­­­­­­های تغذیه ای زنان انجام شد.   روش بررسی: در این پژوهش نیمه تجربی، 108 نفر از زنان مراجعه کننده به مراکز بهداشتی درمانی شهرستان ارومیه در تحقیق وارد. از بین مراکز بهداشتی درمانی شهرستان ارومیه 4 مرکز به طور تصادفی انتخاب گردید که دو مرکز به آموزش از طریق همتا و دو مرکز به آموزش از طریق پرسنل بهداشتی اختصاص داده شد. از بین زنان 35-18 تحت پوشش مراکز منتخب، تعداد 27 نفر به طور تصادفی، در تحقیق وارد شدند. ابزار جمع آوری اطلاعات، پرسشنامه از پیش ساخته شده ای بود که روایی و پایایی آن مورد تایید قرار گرفته بود. ابتدا از هر دو گروه پیش آزمون به عمل آمد. در گروه آموزش توسط همتایان در هر مرکز 2 نفر از کسانی که بالاترین نمره را کسب نمودند، به عنوان آموزش دهنده همتا انتخاب شدند. گروه همتایان توسط آموزش دهنده های همتا وگروه پرسنل توسط پرسنل بهداشتی 4 جلسه آموزشی 2ساعته را دریافت کردند.6 هفته پس از پایان جلسات آموزشی پس آزمون به عمل آمد. تجزیه وتحلیل آماری با استفاده از آزمون T مستقل و T زوجی انجام شد.   یافته ها: قبل از انجام مداخله میانگین نمرات آگاهی، نگرش و شاخصهای جهانی تغذیه( Global Dietary Index )، مصرف چربی( Fat Consumption Index ) و مصرف گوشت( Meat Consumption Index ) در گروه پرسنل بهداشتی به ترتیب 48/25، 68/6، 77/7، 38/5، 11/2 ودر گروه همتایان 38/30، 20/8، 78/7، 75/5، 33/2بود که پس از انجام مداخله آموزشی در گروه پرسنل بهداشتی به ترتیب به 83/31، 88/8، 25/7، 9/4، 2 ودر گروه همتایان به 4/34، 4/15، 33/5، 62/3، 85/0 تغییر یافت. اختلاف معنی دار آماری بین میانگین نمره آگاهی، نگرش و شاخصهای تغذیه ای دو گروه بعد از آموزش وجود داشت، افزایش میانگین نمرات آگاهی افراد گروه آموزشی پرسنل بیشتر از گروه آموزشی همتایان بود. همچنین میانگین نمره نگرش در گروه همتا بعد از مداخله به طور معنی داری بیش از گروه پرسنل افزایش یافت و نیز بهبود شاخصهای جهانی تغذیه، مصرف چربی و مصرف گوشت در گروه آموزشی همتا بعد از مداخله به طور معنی داری بیش از گروه پرسنل بود.   نتیجه گیری: افزایش بیشتر نمرات آگاهی در گروه پرسنل می تواند نشان دهنده ضعف همتایان در ارائه مطالب نظری وعلمی باشد. اثربخشی آموزش تغذیه بمنظور بهبود شاخصهای تغذیه­ای و نگرش در زنان توسط همتایان بیشتر از پرسنل بهداشتی است، لذا پیشنهاد می گردد این شیوه آموزشی، بخصوص در آموزش تغذیه بیشتر مورد استفاده قرار گیرد.

Title: بررسی تاثیر استراتژی های مدیریت دانش بر نوآوری و عملکرد سازمانی

http://tbj.ssu.ac.ir/article-1-91-fa.html

سابقه و اهداف: عملکرد سازمانها به ویژه در بخش بهداشت و درمان مبنای توسعه کمی و کیفی هر کشور محسوب می گردد و مادامی که سازمانها در زمینه ارزیابی دارایی های دانشی خود، پیمایشی انجام ندهند، مدیریت دانش منجر به بهبود عملکرد و توسعه سازمانی نمی گردد.این تحقیق به بررسی تاثیر مدیریت دانش استراتژیک بر نوآوری و عملکرد سازمانی در مراکز بهداشتی و درمانی می پردازد.   روش بررسی: این تحقیق از لحاظ هدف کاربردی، از نظر روش گردآوری داده ها از نوع توصیفی- پیمایشی است. جامعه آماری این تحقیق مراکز بهداشتی و درمانی شمال فارس می باشد. در این تحقیق 30 برابر متغیر های تحقیق که 120 نفر می باشد به عنوان نمونه آماری انتخاب گردید.برای تست فرضیات این تحقیق از پرسشنامه ای مشتمل بر 20 سوال بسته استفاده گردید. به منظور بررسی اعتبار سازه با استفاده از نرم افزار لیزرل از تحلیل عاملی تأییدی استفاده شد و به منظور تجزیه و تحلیل یافته‎ها از آزمون معادلات ساختاری استفاده گردید.   یافته ها: به منظور بررسی تاثیر کد گذاری دانش و شخصی سازی دانش بر نوآوری و عملکرد، رابطه متغیر ها به صورت مستقیم مورد بررسی قرار گرفت. مقادیر t متناظر با مقادیر استاندارد شده مدل مسیر نهایی نشان می دهد که با بیش از 99 درصد اطمینان متغیرهای نوآوری و عملکرد سازمانی با کد گذاری رابطه مستقیم دارند.همچنین نوآوری و عملکرد سازمانی نیز با 99 درصد اطمینان با شخصی سازی دانش رابطه مستقیم دارد. همچنین مقادیر t متناظر با مقادیر استاندارد نشان می دهد که نوآوری با 99 درصد اطمینان با عملکرد سازمانی رابطه مستقیم دارد.   نتیجه گیری: نتایج تحقیق نشان می دهد که شخصی سازی دانش و کد گذاری دانش تاثیر مثبتی بر نوآوری و عملکرد سازمانی دارد و همچنین این متغیر ها از طریق نوآوری بر عملکرد سازمانی تاثیر مثبتی می گذارند و بین نوآوری و عملکرد سازمانی نیز رابطه مثبت و معنی داری وجود دارد.

Title: بررسی میزان شیوع اختلالات اسکلتی - عضلانی ناشی از کار در کارگران یک صنعت قطعه سازی خودرو

http://tbj.ssu.ac.ir/article-1-92-fa.html

سابقه و اهداف: اختلالات اسکلتی- عضلانی مرتبط با کار عمدتا ناشی از وضعیت های بدنی نامناسب، بلند کردن و حمل دستی بار، حرکات تکراری و عدم طراحی مناسب فضا و پست های کاری می باشند، بنابراین مطالعه حاضر با هدف بررسی میزان شیوع اختلالات اسکلتی- عضلانی ناشی از کار در کارگران یک صنعت قطعه سازی خودرو انجام گردید. روش بررسی: در یک مطالعه توصیفی- تحلیلی و به روش مقطعی(Cross-sectional) ، میزان شیوع اختلالات اسکلتی- عضلانی ناشی از کار 1383 نفر از کارگران دو مجتمع از بزرگترین صنعت قطعه سازی خودرو کشور با استفاده از پرسشنامه استاندارد نوردیک مورد مطالعه و بررسی قرار گرفت. یافته‌ها: بر اساس نتایج حاصل از مطالعه، 2/81 درصد از کارگران مورد بررسی اظهار نمودند که طی یک سال گذشته، حداقل در یکی از نواحی 9 گانه بدن خود، دچار اختلالات اسکلتی- عضلانی بوده اند، به طوری که بیشترین اختلالات به ترتیب مربوط به ناحیه کمر (8/61 درصد) ، دست/مچ دست (2/59 درصد) و آرنج (7/48 درصد) می باشد. نتیجه گیری: نتایج حاصل از این مطالعه نشان می دهد که میزان شیوع اختلالات اسکلتی- عضلانی ناشی از کار در کارگران صنعت قطعه سازی خودرو مورد مطالعه، بسیار بالا می باشد. لذا جهت بهبود شرایط کار، اجرای برنامه های پیشگیری از وقوع این اختلالات و طراحی ارگونومیک محیط کار، شکلی کاملا ضروری به خود می گیرد.

Title: مکان‌یابی محل دفن پسماند شهری با استفاده ازمدلAHP_FUZZY در محیط GIS

http://tbj.ssu.ac.ir/article-1-93-fa.html

سابقه و اهداف: مکان‌یابی جایگاه‌های مناسب دفن پسماند از اقدامات مهم در فرآیند مدیریت جامع پسماندهای جامد شهری است. در فرآیند مکان‌یابی می‌بایست معیارهای مختلفی مورد ملاحظه قرار ‌گیرند. بنابراین کاربرد روش‌هایی که قادر به تامین معیارهای مورد نظر باشند، ضروری است.   روش بررسی: این تحقیق با هدف مکان‌یابی زیست محیطی محل دفن پسماند‌های شهر شیراز با استفاده از روش تحلیل سلسله مراتبی، منطق فازی و بهره‌گیری از نرم افزار Expert Choice انجام گرفته است. به همین منظور، در مرحله اول، عوامل موثر در مکان یابی محل دفن مواد زاید شناسایی شدند. سپس با رقومی کردن و وزندهی 19 لایه بر اساس استانداردهای موجود در نهایت نسبت توافق ( CR ) محاسبه می‌شود، اگر نسبت توافق کمتر از 1/0 بدست آید مقایسات زوجی صحیح و وزن‌های محاسبه شده قابل استفاده می‌باشد.   یافته ‌ها: در این پژوهش با استفاده از داده‌هایی چون فاصله از محدوده قانونی شهر، فاصله از جاده، فرودگاه، کاربری اراضی، قابلیت اراضی، عوارض مصنوع (روستا، تاسیسات و تجهیزات شهری، معادن، ...)، گسل، روند توسعه فیزیکی شهر شیراز، آب‌های سطحی، جهت باد، تراکم جمعیتی، خاکشناسی، هیپستومتریک (طبقات ارتفاعی)، شیب، جهت شیب، پوشش گیاهی، تیپ اراضی، زمین شناسی،.... اطلاعات و نقشه‌ها از طریق مدل‌های مختلف بر اساس مدل منطقی ( AHP-Fuzzy ) تلفیق شدند.   نتیجه ‌گیری: در نهایت بعد از ژئورفرنس نمودن این داده‌ها و مدل‌ها با تصاویر ماهوره‌ای در نهایت منطقه‌ای در شعاع 19 کیلومتری شهر شیراز با 272 هکتار مساحت در محدوده شمال که دارای توان پذیرش پسماند به وزن بیش از هزار تن در هر روز به مدت 15 سال را دارا می باشد، مکان گزینی گردید.

Title: روشهای کنترل مخاطرات اخلاقی در نظام سلامت: مداخلات تقاضا و مداخلات عرضه (مقاله مروری)

http://tbj.ssu.ac.ir/article-1-94-fa.html

سابقه و اهداف: مخاطره اخلاقی به تغییر رفتار ارائه کننده خدمت (پزشک) و گیرنده خدمت (بیمار) در نتیجه بیمه بودن اشاره دارد. مخاطره اخلاقی یک نگرانی در بازار بیمه ی سلامت و به کارگیری سیاستهای بیمه ای در اصلاح نظام سلامت است. مخاطرات اخلاقی پیامدهای نامناسبی از جمله کاهش رفاه، افزایش هزینه­های سلامت، کاهش پوشش بیمه ای، مصرف غیر ضروری منابع، کاهش فواید انباشت خطر و کاهش کارایی فنی و تخصیصی را به دنبال دارد. بنابراین ما در این مقاله مروری برآنیم تا به مداخلات سیاستی مناسب برای جلوگیری از مخاطرات اخلاقی مصرف کننده و ارایه کننده خدمت بپردازیم.   روش بررسی: این مقاله یک مطالعه مروری است و مقالات مرتبط با موضوع از متون منتشر شده و سایت های معتبر انتخاب و مورد مطالعه قرار گرفت. پایگاه­های داده­ای Medline ، Web of Science ، Science Direct ، Google Scholar و Springer با استفاده از کلید واژه های مناسب روش های مورد استفاده در کنترل مخاطرات اخلاقی در بعد تقاضا و عرضه ی خدمت مورد بررسی قرار گرفت.   یافته­ها: سیاستهای طرف تقاضا به تنهایی فقط روی تقاضای خدمات از سوی بیمار تاثیر می گذارد و مانع ارایه خدمات اضافی از سوی ارایه کننده نمی شود. بنابراین تلاش­های بیشتر از طریق سیاستهای طرف عرضه نیز لازم است تا مصرف خدمات اضافی را کنترل کند. در بعد تقاضا از روش هایی مانند فرانشیز، پرداخت ثابت، صف انتظار و تشویق عدم استفاده، و در بعد عرضه از انگیزه های مالی و روش­های پرداخت (کارانه، پرداخت گلوبال)، اصلاح ساختاری نظام ارایه خدمت و مداخلات رفتاری (ارجاع انحصاری و gate-keeping ، استفاده از راهنماهای بالینی) و نظارت های آینده نگر بر نرخ استفاده (انجام آزمایش قبل از پذیرش، گرفتن پیش تاییدیه) برای کاهش مخاطرات اخلاقی استفاده می شود.   نتیجه گیری: راه­های مختلفی که برای کنترل خطرات اخلاقی استفاده می­شوند ممکن است با جلوگیری از مصرف منابع برای خدمات کم ارزش، غیر ضروری و سوء تخصیص منابع، کارایی را ارتقا بخشند و افزایش هزینه­های بخش سلامت را کنترل کنند.    مخاطره اخلاقی، کنترل مخاطرات اخلاقی طرف عرضه، کنترل مخاطرات اخلاقی طرف تقاضا
